# Isolation of Infective Zika Virus from Urine and Saliva of Patients in Brazil

**DOI:** 10.1371/journal.pntd.0004816

**Published:** 2016-06-24

**Authors:** Myrna C. Bonaldo, Ieda P. Ribeiro, Noemia S. Lima, Alexandre A. C. dos Santos, Lidiane S. R. Menezes, Stephanie O. D. da Cruz, Iasmim S. de Mello, Nathália D. Furtado, Elaine E. de Moura, Luana Damasceno, Kely A. B. da Silva, Marcia G. de Castro, Alexandra L. Gerber, Luiz G. P. de Almeida, Ricardo Lourenço-de-Oliveira, Ana Tereza R. Vasconcelos, Patrícia Brasil

**Affiliations:** 1 Laboratório de Biologia Molecular de Flavivírus, Instituto Oswaldo Cruz, Fiocruz, Rio de Janeiro, Brazil; 2 Laboratório de Doenças Febris Agudas, Instituto Nacional de Infectologia Evandro Chagas, Fiocruz, Rio de Janeiro, Brazil; 3 Laboratório de Mosquitos Transmissores de Hematozoários, Instituto Oswaldo Cruz, Fiocruz, Rio de Janeiro, Brazil; 4 Laboratório Nacional de Computação Científica, Petrópolis, Rio de Janeiro, Brazil; University of Rhode Island, UNITED STATES

## Abstract

**Background:**

Zika virus (ZIKV) is an emergent threat provoking a worldwide explosive outbreak. Since January 2015, 41 countries reported autochthonous cases. In Brazil, an increase in Guillain-Barré syndrome and microcephaly cases was linked to ZIKV infections. A recent report describing low experimental transmission efficiency of its main putative vector, *Ae*. *aegypti*, in conjunction with apparent sexual transmission notifications, prompted the investigation of other potential sources of viral dissemination. Urine and saliva have been previously established as useful tools in ZIKV diagnosis. Here, we described the presence and isolation of infectious ZIKV particles from saliva and urine of acute phase patients in the Rio de Janeiro state, Brazil.

**Methodology/Principal Findings:**

Nine urine and five saliva samples from nine patients from Rio de Janeiro presenting rash and other typical Zika acute phase symptoms were inoculated in Vero cell culture and submitted to specific ZIKV RNA detection and quantification through, respectively, NAT-Zika, RT-PCR and RT-qPCR. Two ZIKV isolates were achieved, one from urine and one from saliva specimens. ZIKV nucleic acid was identified by all methods in four patients. Whenever both urine and saliva samples were available from the same patient, urine viral loads were higher, corroborating the general sense that it is a better source for ZIKV molecular diagnostic. In spite of this, from the two isolated strains, each from one patient, only one derived from urine, suggesting that other factors, like the acidic nature of this fluid, might interfere with virion infectivity. The complete genome of both ZIKV isolates was obtained. Phylogenetic analysis revealed similarity with strains previously isolated during the South America outbreak.

**Conclusions/Significance:**

The detection of infectious ZIKV particles in urine and saliva of patients during the acute phase may represent a critical factor in the spread of virus. The epidemiological relevance of this finding, regarding the contribution of alternative non-vectorial ZIKV transmission routes, needs further investigation.

## Author Summary

The American continent has recently been the scene of a devastating epidemic of Zika virus and its severe manifestations, such as microcephaly in newborns and Guillain-Barré Syndrome. Zika virus, first detected in 1947 in Africa, only from 2007 started provoking outbreaks. Zika, dengue and chikungunya viruses are primarily transmitted by *Aedes* mosquitoes. Dengue has been endemic in Brazil for almost 30 years, and the country is largely infested by its main vector, *Aedes aegypti*. Chikungunya virus entered the country in late 2014 and Zika presence was confirmed eight months later. Nevertheless, Zika notifications multiplied and spread across the country with unprecedented speed, raising the possibility of other transmission routes. This hypothesis was strengthened by some recent reports of Zika sexual transmission in *Ae*. *aegypti*-free areas and by the description of a low transmission efficiency to Zika virus in local *Ae*. *aegypti*. We found Zika active particles in both urine and saliva of acute phase patients, and a finding that was promptly announced by Fiocruz via Press Conference on February 5, 2016. In this work, we bring up the potential alternative person-to-person infection routes beyond the vectorial transmission, that might have epidemiological relevance.

## Introduction

Zika virus (ZIKV) is an emerging mosquito-borne virus of the family *Flaviviridae* and genus *Flavivirus* [1]. ZIKV was first reported in 1947 after isolation from a febrile sentinel rhesus monkey [2]. Since then, serologic evidence of human ZIKV infection in Africa and Asia was detected, but until 2005 only few human cases were reported [3]. The first well-described outbreak outside these geographic regions happened in 2007 in Micronesia, more specifically in Yap State, when the majority of the population was affected with Zika fever [4]. Intriguingly, the local mosquito vector was not confirmed by neither viral isolation nor molecular methods [4].

On October 2013, a second intense outbreak in Oceania occurred in French Polynesia (2013/2014), and soon after spread over to New Caledonia (2014), Cook Islands, (2014) and Easter Island, 2014 [5, 6]. In these outbreaks, approximately 80% of ZIKV infections were asymptomatic [4, 7]. Commonly, Zika is considered to be a mild disease lasting one week with symptoms including fever, rash, conjunctivitis, arthralgia, myalgia, headache and malaise. However, during the French Polynesian epidemic, its association with severe neurological complications, the Guillain-Barré syndrome (GBS) was reported for the first time [8].

In April 2015, the first autochthonous cases in the Americas were identified in Brazil [9, 10]. At present, Brazil is suffering from an explosive outbreak of ZIKV. Hence, in February 2016, Brazilian Ministry of Health (MoH) appraised the incidence of greater than one million cases of ZIKV disease cases [11]. Notably, in addition of an increase of GBS cases as occurred in the French Polynesia outbreak, the MoH of Brazil described a rise of microcephaly occurrence. Between 22 October 2015 to 5 March 2016, 6158 cases of microcephaly and/or central nervous system malformation were noticed in contrast to the estimated average number of 163 annual cases [12]. So far, 745 suspected cases of microcephaly have been confirmed as ZIKV-associated microcephaly in a total of 1927 investigated cases [11–13]. More recently, a case of ZIKV infection with vertical transmission demonstrated the association of severe fetal brain injury with fetal infection with ZIKV [14]. Moreover, ZIKV nucleic acid was detected in amniotic fluid of two pregnant women, whose fetuses were diagnosed with microcephaly, corroborating vertical transmission possibility [15]. Other abnormalities such as placental insufficiency, fetal growth restriction, CNS injury, and fetal death have also been reported in association with ZIKV infection [16]. This scenario of ZIKV infection linked to severe neurological complications as well as the establishment of ongoing ZIKV outbreaks in several countries in Latin America led to the WHO to declare ZIKV an international public health emergency [11, 17, 18].

The transmission of ZIKV has been associated with several *Aedes* mosquito species belonging to subgenus *Stegomyia*, notably *Ae*. *aegypti* [19, 20] and *Ae*. *albopictus* [21]. However, a recent study proposes that although susceptible to infection, *Ae*. *aegypti* and *Ae*. *albopictus* from the Americas display an unexpectedly low vector competence for a fifth-passage ZIKV strain from New Caledonia [22], suggesting other factors such as the large naïve population for ZIKV and the high densities of human-biting mosquitoes contribute to the rapid spread of ZIKV during the current outbreak. Nonetheless, perinatal transmission [23] and potential risk for transfusion-transmitted ZIKV infections has also been demonstrated [24]. Most remarkably, ZIKV can be likely disseminated by sexual contact, due to its presence in semen [25, 26]. In addition, it was demonstrated that ZIKV exists in urine [27, 28], breast milk [29] and saliva [30]. Indeed, ZIKV was more frequently detected in urine and saliva than in blood using ZIKV RT-PCR tests for diagnosis. It was considered that patients exhibit the highest concentrations of ZIKV in saliva at disease onset [30] while in urine, ZIKV possibly remains detectable for longer periods [27]. In this study, we demonstrate that it is possible to recover infective ZIKV from both saliva and urine of acute phase patients by means of viral isolation in Vero cells. This achievement suggest that ZIKV may be transmitted between humans by infected saliva and urine.

## Methods

### Ethics statement

The Acute Febrile Illnesses Laboratory and Molecular Biology of Flavivirus Laboratory conducted this study at Oswaldo Cruz Foundation, Rio de Janeiro. The institutional review boards at Fundação Oswaldo Cruz (Fiocruz) approved the study protocol. All subjects provided written, informed consent before participation, and a medical assistant filled a standardized medical questionnaire form, during an interview with the participants.

### Study facilities and patients enrollment

In this study, most enrolled patients (6 out of nine) were selected from the cohort of pregnant women with rash [16], with exception of two men and one woman who went to the consultation in the non-pregnant branch of the Acute-Fever Illnesses Clinic of Fiocruz [31]. The age distribution of the nine patients is consistent with the overall age profile of the clinic. The inclusion criteria was based on the presence of pruritus/itching rash as they were identified as symptoms that can potentially help in distinguishing ZIKV from other arboviral infections [31]. A standard case report form was utilized to record information about demographics and clinical features. Urine and saliva samples were asked for all the enrolled patients, but four patients did not managed to collect saliva. Numbers of days from the first reported symptom (days after symptoms onset) and main signs and symptoms were recorded. Urine and saliva samples investigated in this study were collected from January 14th to February 2nd, 2016.

### Clinical samples

Saliva and urine specimens were collected in 50 mL sterile certified, DNase-/RNase-free tubes, and after collection, in some cases, the pH was measured by a digital pH meter, in order to investigate the relevance of the pH for viral infection. Twenty five millimeter diameter sterile syringe filters with a 0.22 μm pore size were used to filter the specimens. The samples were aliquoted for subsequently analysis and assays, as infection in Vero cell culture and RNA isolation.

### Primary viral isolation

The African green monkey kidney (Vero) cell line (ATCC- CCL81) was grown in 37°C, under an atmosphere containing 5% CO_2_, in Earle’s 199 medium supplemented with 5% fetal bovine serum (FBS) and 40 μg/ml of gentamicin. The Vero cells were seeded at a density of 40,000 cells/cm^2^ in 25 cm^2^ culture flasks 24 hours before inoculation. The urine and saliva samples were diluted in Earle’s 199 medium supplemented with 5% FBS (1:2 and 1:4), and 1 mL of each dilution was inoculated onto Vero cells monolayer. After 1 h incubation at 37°C, the inoculum was removed and replaced by 10 mL culture medium in the presence of 40 μg/ml of gentamicin. As negative control for each experiment, Vero cells seeded in one culture flask were mock inoculated with culture media. The presence of infectious viral particles was controlled by observation of cytopathic effects (CPE).

### Plaque forming unit assay

Vero cells were seeded at a density of 40,000 cells/cm^2^ in 6-well plates 24 h before inoculation. Dilutions of the biological specimens (1:2, 1:4 and 1:8) in culture media were used to infect monolayers (200 μL/well). After 1 h incubation at 37°C, the inoculum was removed and replaced by 3 mL of 2.4% CMC (carboxymethyl cellulose) in Earle’s 199 medium. After 7 days incubation at 37°C, cells were fixed with 10% formaldehyde, washed, and stained with 0.4% crystal violet for visualization of plaques.

### RNA isolation

Viral RNA was isolated from 140 μL of each biological specimens and cell culture supernatant using the QIAamp Viral RNA Mini Kit (Qiagen, Hilden, Germany) according to the manufacturer’s recommendations. RNA was eluted in 60 μl of AVE buffer and stored at -80°C until use. The concentration and purity of each RNA sample were measured by Thermo Scientific NanoDrop 8000 Spectrophotometer and Agilent 2100 Bioanalyzer using the Agilent RNA 6000 Nano Kit according the manufacturer’s instructions.

### RT-PCR

The viral RNA was reverse transcribed applying the Superscript IV First-Strand Synthesis System (Invitrogen) using random hexamers according to the manufacturer’s recommendations. The reverse transcription reaction was carried out at 23°C for 10 min, 55°C for 10 min and 80°C for 10 min. Further, the viral RNA was amplified by conventional PCR using GoTaq Green Master Mix (Promega) according to the manufacturer’s recommendations. The set of primers utilized in this procedure were: ZK3F, 5' GCTACTGGATTGAGAGTGAGAAG 3', and ZK2R, 5' CTCAGAGATGGTCCTCTTGTTC 3´ for ZIKV; CHIK E1 F, 5´TACCCATTCATGTGGGGC3´ and CHIK E1R, 5´GCCTTTGTACACCACGATT 3´ [32]; and DEN F, 5´ TCAATATGCTGAAACGCG CGAGAAACCG 3´ and DEN R, 5´ TTGCACCAACAGTCAATGTCTTCAGGTTC3´ for DENV [33]. The thermocycling program set up in a Veriti 96 Well thermocycler (Applied Biosystem) was 1 cycle of 95°C for 5 min; 40 cycles of 95°C for 40 sec, 50°C for 40 sec, 72°C for 30 sec; 1 cycle of 72°C for 10min and hold of 4°C. 10 ml of Amplified products were detected by electrophoresis on a 2% agarose gel, visualized by ethidium bromide staining UV.

### NAT and quantitative RT-PCR

To discard co-infection of ZIKV with dengue and/or chikungunya viruses, we analyzed the urine, saliva samples and the viral strains isolated from Vero cell using he NAT- Dengue, Zika and Chikungunya discriminatory kit (Instituto de Biologia Molecular do Paraná and Fundação Oswaldo Cruz, Brazil). To measure genomic ZIKV load, viral RNA was reverse transcribed and amplified using the TaqMan Fast Virus 1-Step Master Mix (Applied Biosystems) in an Applied Biosystems StepOnePlus Instrument. For each reaction we used 400 nM forward primer (5’-CTTGGAGTGCTTGTGATT-3’, genome position 3451–3468), 600 nM reverse primer (5’-CTCCTCCAGTGTTCATTT-3’, genome position 3637–3620) and 250 nM probe (5’FAM- AGAAGAGAATGACCACAAAGATCA-3’TAMRA, genome position 3494–3517). The sequences of this primer set were kindly provided by Isabelle Lepark-Goffart (French National Reference Centre for Arboviruses, IRBA, Marseille, France). Samples were run in duplicate. The reverse transcription was performed at 50°C for 5 minutes. The qPCR conditions were 95°C for 20 seconds, followed by 40 amplification cycles of 95°C for 15 seconds and 60°C for 1 minute. Copy numbers of ZIKV genomic RNA were calculated by absolute quantitation using a standard curve for each run. To construct a standard curve, we cloned an amplicon comprising the genomic region 3085–4032 of the isolate Rio-U1 using pGEM-T Easy Vector (Promega) to serve as a template for in vitro transcription. The RNA transcript was made with mMessage mMachine High Yield Capped RNA Transcription Kit (Invitrogen) using T7 enzyme and purified using MEGAclear Kit (Ambion) according to manufacturer’s instructions. The purity of the transcript was verified using NanoDrop 8000 Spectrophotometer (Thermo Scientific), the integrity was analyzed using 2100 Bioanalyzer (Agilent) using the RNA 6000 Nano Kit (Agilent), and the concentration of the RNA was accessed using Qubit 2.0 Fluorometer (Invitrogen). The standard curve was generated by a ten-fold dilution (ranging from 10 to 10^9^ copies/reaction) of the transcript. The limit of detection under standard assay conditions was approximately 40 viral RNA copies/mL.

### Nucleotide sequence

Viral RNA samples were obtained from the first passage of Vero cell isolates from urine of patient 1 and saliva of patient 6. Double-stranded cDNA libraries were prepared using the TruSeq Stranded mRNA LT Sample Preparation Kit (Illumina, San Diego, CA, USA). Briefly, the polyA containing mRNA purification step was not performed and the protocol was started with 25–35 ng of RNA in 5 ul of molecular biology grade water to which were added 13 ul of Fragment, Prime, Finish Mix. The remaining steps of the protocol were carried out without any modifications. Library quality control was performed using the 2100 Bioanalyzer System with the Agilent DNA 1000 Kit (Agilent, Santa Clara, CA, USA). The libraries were individually quantified via qPCR using a KAPA Library Quantification Kits for Illumina platforms (KAPA Biosystems, Wilmington, MA, USA). The libraries were pooled together in equimolar quantities and sequenced. Paired-end reads (2 × 75 bp) were obtained using a MiSeq Reagent Kits v3 (150-cycles) in a MiSeq sequencing system (Illumina).

### Assembly and annotation

A total of 17,413,830 reads was generated for Rio-U1 sample and 21,734,486 for Rio-S1 sample. Related reads to *Chlorocebus sabaeus* have been filtered using Bowtie2 and Samtools, remaining 12,614,062 reads of Rio-U1 and 12,943,134 of Rio-S1. Both genomes were assembled using Ray 2.20 (k = 31). The completed genome of Rio-U1 has 10,795bp (Accession number KU926309) and Rio-S1 has 10,805bp (Accession number KU926310). Gene prediction was performed by GenemarkS 4.17. Mature peptides were identified by blastp against the protein annotated in reference sequence NC_012532.

### Phylogenetic analysis

Nucleotide sequences encoding the precursor polyprotein of 39 ZIKV strains and 1 of DENV 4 were aligned using the Clustal W [34]. Evolutionary analysis was performed as described elsewhere [15]. Phylogenetic studies were carried out using the Maximum Likelihood method based on the General Time Reversible model [35] of the MEGA7 software [36]. Evolutionary history of these sequences was represented by bootstrap consensus tree (from 1000 replicates), in a traditional branch style. Branches corresponding to partitions reproduced in less than 50% bootstrap replicates are collapsed. Initial trees for the heuristic search were obtained automatically by applying Neighbor-Join and BioNJ algorithms to a matrix of pairwise distances estimated using the Maximum Composite Likelihood (MCL) approach, and then selecting the topology with superior log likelihood value. A discrete Gamma distribution was used to model evolutionary rate differences among sites (5 categories; +G, parameter = 0.9645). The rate variation model allowed some sites to be evolutionarily invariable ([+I], 37.8665% sites). The analysis involved 40 nucleotide sequences. All positions with less than 95% site coverage were eliminated. There were 10247 positions in the final dataset. Timetree was inferred by Reltime method [37] from MEGA, using GTR (G+I, 5 categories), partial deletion with site coverage cutoff of 95%.

## Results

### Patients, clinical and social demographic characteristics

We examined nine enrolled patients suspected of ZIKV infection. The initial medical support and collection of urine and saliva samples were performed from January 14^th^ to February 2^nd^ 2016. Out of seven women, six were pregnant with gestational ages varying from 18 to 33 weeks, median value of 20.5 ± 5.8 weeks (Table A and B in S1 Text). The female patient ages ranged from 20 to 42 years old (median value of 28.5 ±7.4 years) and the male patient ages were 24 and 68 years old. All the patients live in the metropolitan area of Rio de Janeiro (Table C and D in S1 Text).

The most frequent sign of ZIKV disease was pruritic maculo papular rash which lasted in average 4 days (Table A and B in S1 Text). However, other clinical symptoms were also prevalent, such as low-grade fever (< 38°C), headache, myalgia and arthralgia of large and small joints, present in 5 out of 9 patients.

**Fig 1:**
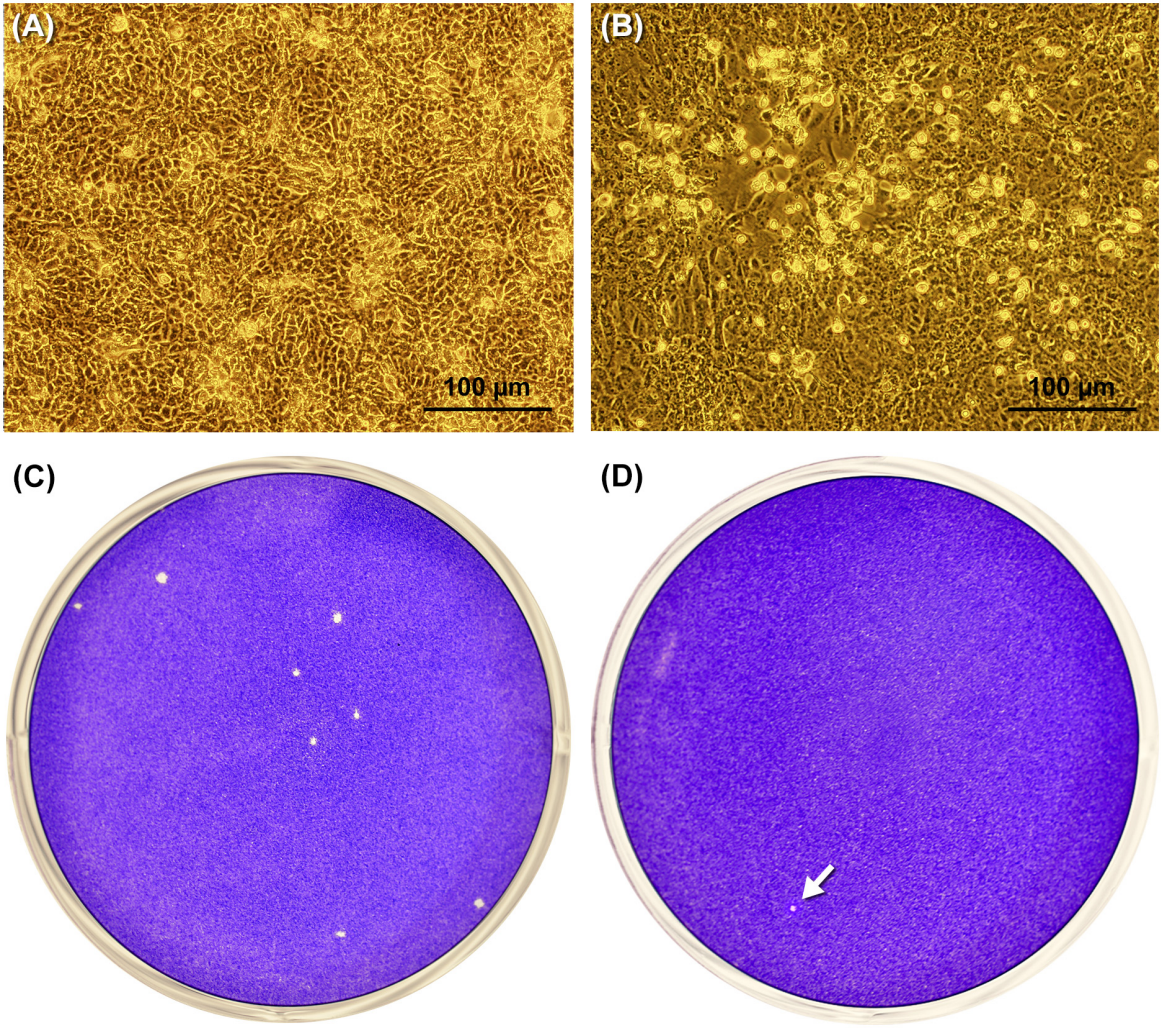
Isolation of Zika virus in Vero cell from the saliva of patient 6. Phase contrast optical microscopy of culture flasks containing (A) Mock-infected Vero cells and (B) saliva-infected Vero cells presenting a clear visible cytopathic effect. Viral plaque detection in saliva (C) and urine (D). The white arrow shows the unique viral plaque detected in the urine sample.

We collected and analyzed urine from patients 1 to 4 and both urine and saliva samples from patients 5 to 9. Vero cells cultures were inoculated at the same date of sample collection and then daily observed through inverted microscopic examination until the appearance of cytopathic effect (CPE). Within one week of incubation, only two samples exhibited CPE (2 out of 14), the urine sample of patient 1 with CPE detected at 4^th^ day of post-inoculation (1 out of 9) and the saliva sample of patient 6 at 5^th^ day post-inoculation (1 out of 5). In this last infection, we recognized small foci of rounded and refractive cells detaching from the monolayer (Fig 1A and 1B). After one-week incubation, we proceeded to split cells from negative cultures by means of trypsinization when monolayer was confluent. This procedure was repeated for three consecutive times. Nevertheless, it was not possible to isolate ZIKV in these samples, neither by detecting CPE in Vero cell monolayers or ZIKV genome by RT-PCR. We also analyzed these samples by plaque forming assay as a way to detect infectious virus particles. Unfortunately, we did not perform this analysis with urine of patient 1, because we received a small aliquot of this specimen. Nevertheless, we detected viral plaques from samples of patient 6 (Fig 1C and 1D), in which the dilution 1:2 of saliva originated in 8 PFU resulting in an original viral concentration of 80 PFU/ml in saliva of patient 6. Interestingly, only one viral plaque was visualized by means of this methodology in urine sample of this patient 6, resulting in a titer of 10 PFU/ml.

### ZIKV diagnosis and genome detection

**Fig 2:**
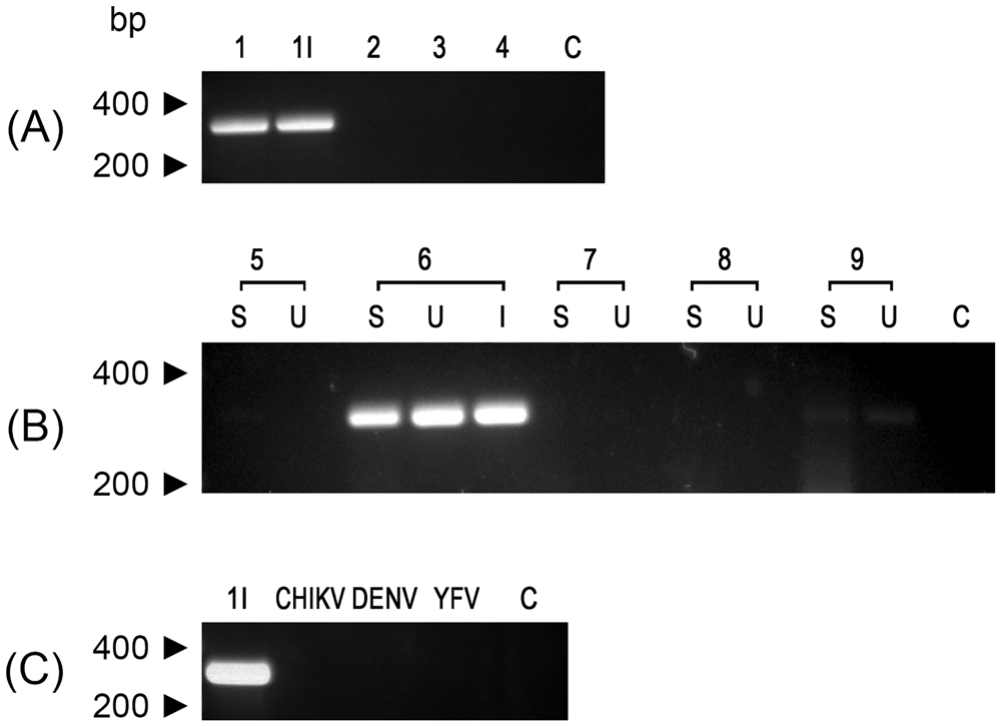
Detection of genomic RNA of Zika virus in urine and saliva samples by RT-PCR analysis. (A) Shows the profiles obtained from urine samples. The lane numbers indicate the patient code. The lane 1I is the amplicon obtained from the viral isolate from urine of patient 1 (isolate Rio-U1). (B) RT-PCR analysis from patients 5 to 9 where S indicates saliva RNA samples, U, urine RNA samples, and I viral isolate sample. (C) Amplification of Zika virus genome of isolate Rio-U1 (1I) with ZIKV-specific primers that were also employed in the RT-PCR assay of Chikungunya virus RNA (CHIKV), dengue virus RNA (DENV) and Yellow Fever 17DD RNA (YFV). In all of these analyses, a negative control of amplification were included (C). The size marker migration is indicated on the left of the figures.

Furthermore, we analyzed all urine and saliva specimens by RT-PCR to confirm the detection of ZIKV (Fig 2A and 2B). In addition, we included RNA samples of ZIKV isolated from patient 1 and 6 in Vero cells. The set of samples of patient 1 and 6 were all positive and an expected-amplicon band of around 300 bp was seen in electrophoretic analyses, demonstrating the presence of ZIKV genome in these samples (Fig 2A and 2B). We also observed a faint band from urine and saliva of patient 9 (Fig 2B). The ZIKV specificity of this approach was confirmed when we tested this protocol in RNA samples of Chikungunya (CHIKV), dengue (DENV) and yellow fever (YFV) viruses (Fig 2C).

Notwithstanding, it was mandatory to confirm the result of Zika virus infections in patients and isolations in Vero cells, since ZIKV, DENV and CHIKV are co-circulating in Brazil and the diseases caused by them exhibit similar symptoms. So, each sample was tested for the presence of these three viruses by the ZIKV nucleic acid testing (NAT) of samples which was established to be routinely used in Brazil as diagnosis test since December 2015. (Table 1). All patients included in this study were negative for DENV and CHIKV (Ct > 40.0). Patient 1 was positive for ZIKV in urine (Ct of 30.02) and patient 6 in urine (Ct of 25.56) and saliva (Ct of 30.27) and the viral isolates derived obtained from specimens of these patients were also positive and presented Ct of 12.62 and Ct of 20.88, respectively. Patient 9 was also positive for ZIKV in urine specimen (Table 1), whereas urine from patient 7 presented amplification in a late cycle and, therefore, this result was considered inconclusive. To validate negative results, the ribosomal 18S RNA was detected in all samples showing that there was no inhibition of the RT-PCR.

**Table 1:** ZIKV RNA detection and quantitation.

Patient	Sample	Target	NAT Cт*	Result**	Viral load (vRNA copies/mL)***
1	urine	18 S	24.98	Positive	2,677
		ZIKV	30.02		
	Vero cell	18 S	11.98	Positive	1.24 x 10^10^
		ZIKV	12.62		
2	urine	18 S	19.63	Negative	< 40
		ZIKV	Ud		
3	urine	18 S	21.49	Negative	< 40
		ZIKV	Ud		
4	urine	18 S	24.57	Negative	< 40
		ZIKV	Ud		
5	urine	18 S	24.38	Negative	< 40
		ZIKV	Ud		
	saliva	18 S	22.04	Negative	< 40
		ZIKV	Ud		
6	urine	18 S	21.54	Positive	252,836
		ZIKV	25.56		
	saliva	18 S	17.40	Positive	74,449
		ZIKV	30.27		
	Vero cell	18 S	11.53	Positive	2.88 x 10^9^
		ZIKV	20.88		
7	urine	18 S	24.57	Inconclusive	102
		ZIKV	40.96		
	saliva	18 S	24.02	Negative	< 40
		ZIKV	Ud		
8	urine	18 S	22.89	Negative	< 40
		ZIKV	Ud		
	saliva	18 S	15.35	Negative	< 40
		ZIKV	Ud		
9	urine	18 S	23.41	Positive	431
		ZIKV	37.41		
	saliva	18 S	16.53	Negative	40
		ZIKV	Ud		

* Ud means undetermined (Ct> 40.00).

**Diagnosis of the patients suspected to be infected with Zika virus (NAT- Zika, dengue and chikungunya diagnosis test).

*** Value determined by quantitative RT-PCR.

**Fig 3:**
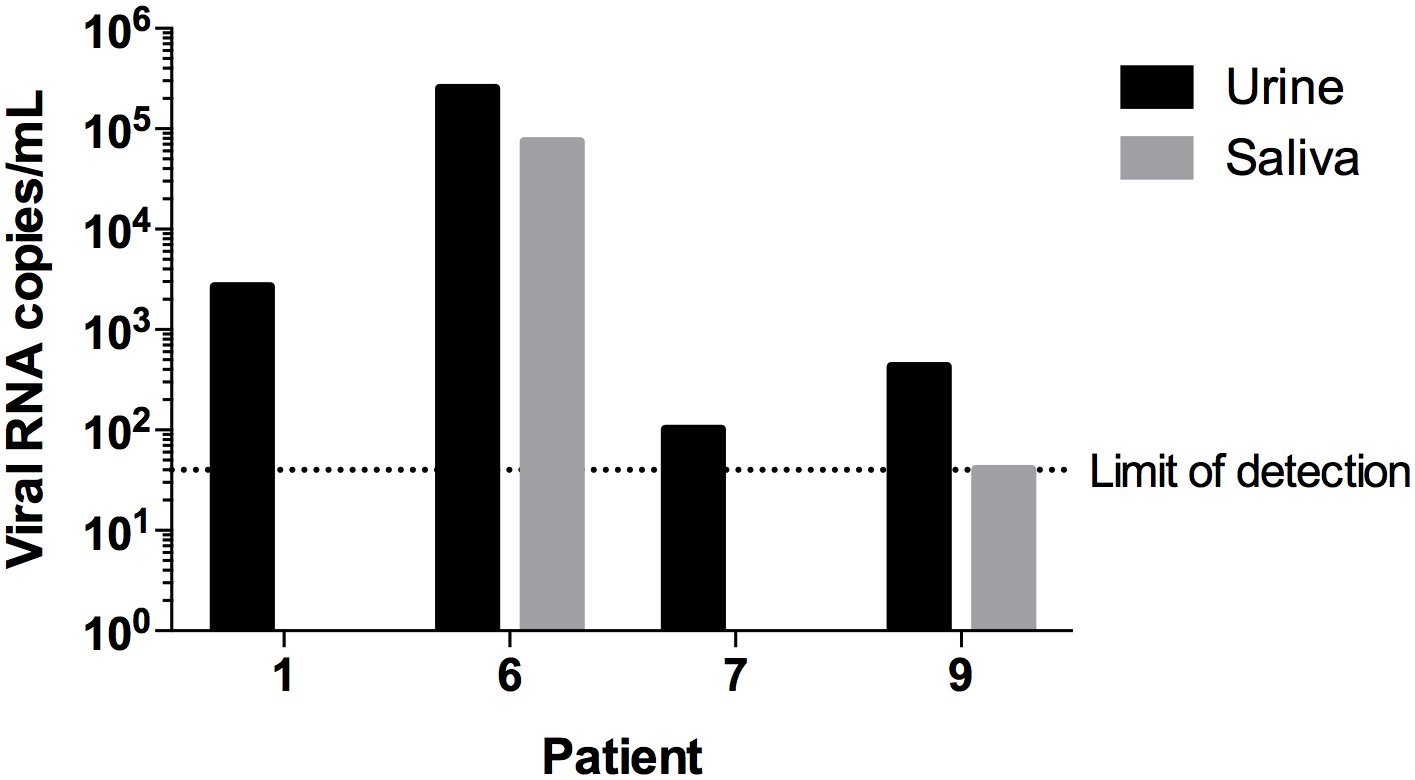
ZIKV Viral loads from urine and saliva specimens of infected patients measured by RT-qPCR. Urine specimens are shown in black and saliva specimens are shown in grey. The limit of detection is shown as a dotted line corresponding to 40 viral RNA copies/mL.

Viral loads of these samples were then measured by a RT-qPCR assay resulting in data consistent with those obtained by the diagnosis assay kit (Table 1 and Fig 3). Accordingly, the highest viral loads were obtained from those specimens that allowed us to isolate ZIKV by Vero cell infections. The urine of patient 1 exhibited a ZIKV-genomic RNA copies of 2.68 x 10^3^ per ml whereas the patient 6 displayed 2.53 x 10^5^ ZIKV RNA copies per ml in urine and 7.44 x 10^4^ ZIKV RNA copies per ml in saliva. As expected for isolated viral samples, we observed an increase of genomic ZIKV RNA copies in Vero-cell- isolated samples, in which the isolated from patient 1 presented 1.24 x 10^10^ copies/ml and patient 6, 2.88 x 10^9^ copies/ml (Fig 3). Furthermore, we confirmed positivity of the urine from patient 7 (102 copies/ml) and the positive detection of ZIKV RNA in saliva (40 copies/ml) and urine (431 copies/ml) of patient 9, although this established value is borderline localized in the limit of detection.

### ZIKV genomic sequencing

**Fig 4:**
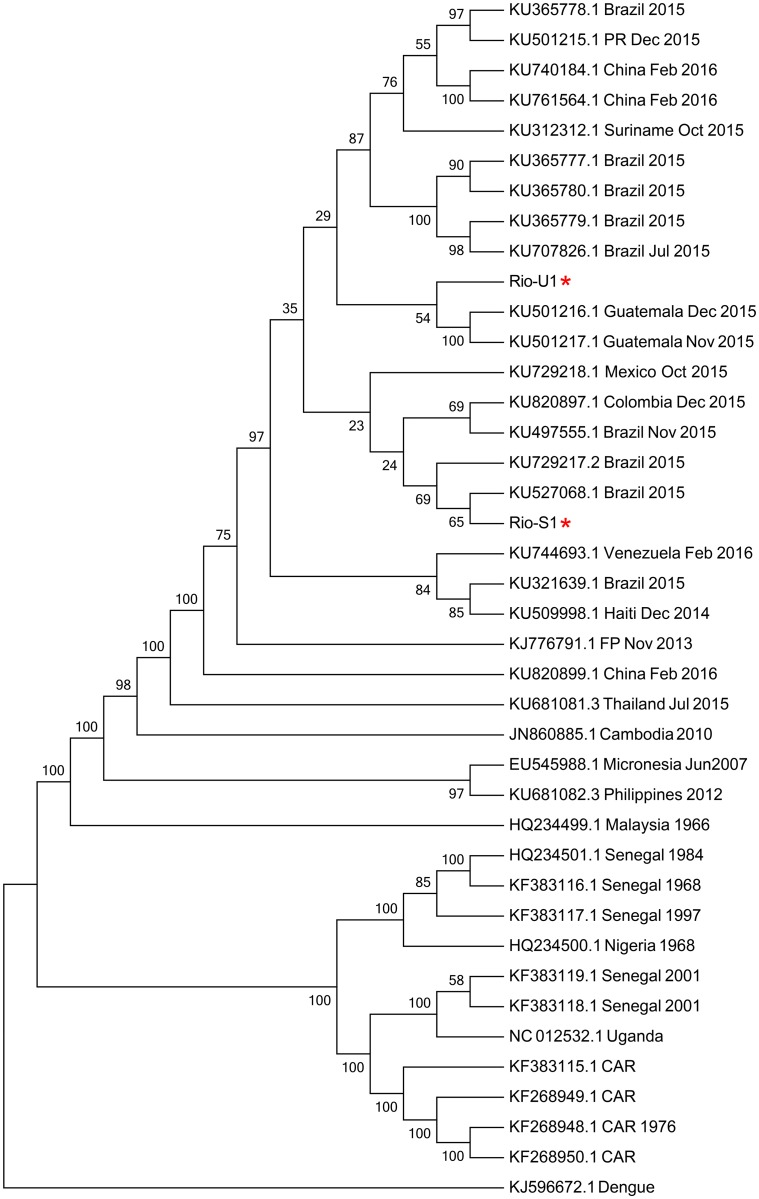
Molecular Phylogenetic analysis by Maximum Likelihood method. The evolutionary history was inferred by using the Maximum Likelihood method based on the General Time Reversible model. The bootstrap consensus tree inferred from 1000 replicates is taken to represent the evolutionary history of the taxa analyzed. Branches corresponding to partitions reproduced in less than 50% bootstrap replicates are collapsed. The percentage of replicate trees in which the associated taxa clustered together in the bootstrap test (1000 replicates) are shown next to the branches. Initial tree(s) for the heuristic search were obtained automatically by applying Neighbor-Join and BioNJ algorithms to a matrix of pairwise distances estimated using the Maximum Composite Likelihood (MCL) approach, and then selecting the topology with superior log likelihood value. A discrete Gamma distribution was used to model evolutionary rate differences among sites (5 categories (+G, parameter = 0.9645)). The rate variation model allowed for some sites to be evolutionarily invariable ([+I], 37.8665% sites). The analysis involved 40 nucleotide sequences. All positions with less than 95% site coverage were eliminated. That is, fewer than 5% alignment gaps, missing data, and ambiguous bases were allowed at any position. There were a total of 10247 positions in the final dataset. Evolutionary analyses were conducted in MEGA7.

The genomic sequences of Vero cell isolates ZIKV Rio-U1 strain (KU926309), isolated from urine and Rio-S1 (KU926310) strain, isolated from saliva, were then determined. The comparison between Rio-U1 and Rio-S1 yielded 99.61% identity, displaying six amino acid variations in the viral proteins (Table 2). For phylogenetic analysis, we used nucleotide sequences coding the complete ZIKV polyprotein. We observed that all sequences sampled in the Americas form a robust monophyletic cluster (bootstrap score = 97%) within the Asian genotype and share a common ancestor with the ZIKV strain that circulated in French Polynesia in November 2013 and remained genetically isolated from African clusters (Fig 4).

**Table 2:** Differences in amino acid residues in ZIKV polyproteins of Rio-S1 and Rio-U1 isolates.

Polyprotein position	ZIKV protein (amino acid position)	Rio-S1	Rio-U1
625	E (335)	A	T
1143	NS1 (349)	V	M
1404	NS2B (32)	M	I
2039	NS3 (537)	K	R
2122	NS4A (3)	T	A
2688	NS5 (168)	A	V

Phylogenetic analysis of the isolated viruses exhibiting the highest identity of ZIKV strain Rio-U1 with KU501216.1 and KU501217.1 both from Guatemala (99.7% identity), isolated also related with the first reported autochthonous transmission of ZIKV in Brazil [38]. Whereas Rio-S1 presented 99.7% of identity with KU527068.1, isolated in Brazil from a Zika-associated microcephaly case [14].

## Discussion

Infective ZIKV particles exists in urine and saliva of patients. We reported this evidence for the first time in a worldwide press release on February 5, 2016. We communicated our data before the Carnival in Brazil (from February 6^th^ to 10^th^), because we were very concerned about the risk of pregnant women to be exposed to ZIKV in an event involving crowds and also considering the global emergency declared by WHO. Part of our discovery, the viral isolation from saliva, was further confirmed in case report study of a patient who developed a febrile illness after returning from the Dominican Republic to Italy [39]. In our study, we also demonstrate the occurrence of infectious Zika viral particles in urine besides of saliva of patients. Moreover, we also showed that the saliva of an acute phase patient may have a viral concentration of 80 PFU/ml. The isolation of two ZIKV samples from urine and saliva was associated with ZIKV load in infected patients during the acute phase. Actually, the presence of ZIKV genome in urine is not a novelty. Hence, former studies preconized the use of urine and saliva for ZIKV RNA detection and diagnosis [27, 30], since ZIKV genome was more frequently identified in saliva and urine compared to blood. Furthermore, the finding of flaviviral genome in urine was earlier described in Dengue [40], Yellow Fever [41], St. Louis Encephalitis [42], Japanese Encephalitis [43], and West Nile viruses [44]. Dengue genome was also detected in saliva of infected patients [40]. Interestingly, the existence of excreted-infectious West Nile particles in the urine of acute phase patients was earlier described in conjunction with their isolation in Vero E6 and in BHK21 cells [45]. Particularly, ZIKV isolation was approached by many groups utilizing Vero cells (GeneBank: KJ776791; JN860885; KU647676). Therefore, we adopted this cell model to detect, amplify and quantify viable ZIKV straight from patient’s samples of urine and saliva.

The recovery of ZIKV from these urine and saliva was effective in two of nine patients whose viral load were clearly detectable. Interestingly, despite the fact that the viral load found in the urine of patient 1 was considerably lower, around one hundred times, than the equivalent sample in patient 6, we only recovered virus from urine of the former (Rio-U1 strain). On the other hand, recovering of infective ZIKV from patient 6, the Rio-S1 strain, was successful using the saliva sample, but not with urine one, even though the highest number of copies has been established in urine. Concordantly, we detected in this analysis a superior number of plaques in plaque assay of saliva. Viral detection and recovery from urine and saliva of ZIKV patients might be firstly related to the severity of infection as well as the period of specimen collection after the onset of Zika symptoms. The detection of ZIKV RNA in saliva improved the diagnosis in the first week from the disease onset [30]. But ZIKV viruria persists for longer periods after disease beginning and, in some cases, for longer than two weeks from Zika onset [27], as described in the two recently reported cases of Guillain–Barré syndrome occurred in Martinica [46]. However, it is necessary to perform additional clinical studies associating disease onset, severity of symptoms and viral persistence in urine and saliva to better clarify this point.

Another aspect in viral recovering deals with the physiological pH found in saliva and urine. Hence, pH in urine varies from 4.5 to 8.0 while saliva assumes values near neutral pH. It is well known that the flavivirus envelope protein E undergoes irreversible conformational changes at a mildly acidic pH (below 6.5), a process naturally occurring in the viral membrane fusion in endosomes [47]. These structural changes are irreversible, and outside of cellular environment, provoke loss of infectivity and hemagglutination activity as well as virus aggregation due to increased hydrophobicity [48]. Thereby, we suggest that the failure of recovering ZIKV strain in Vero cells propagation from the urine of patient 6 would be due to the inactivation of most ZIKV due to exposition of the acidic pH value of 5.6 of this urine specimen. The infectious virus number was lower, at least proportionally to the viral RNA copies presented in this fluid, when compared to saliva of the same patient. We do not establish the pH of patient´s 1 urine, due to volume sample limitations. The importance of ZIKV in urine for human transmission is unexplored, but the effect of acidic pH on viral viability might represent a serious restriction for viral spreading. In West Nile Virus when a similar urine excretion occurs, it is considered that the presence of infectious particles would represent a real risk for inter human transmission through kidney transplantation [45].

In reference to the occurrence of viable ZIKV in saliva, a large range of viruses can be identified in this specimen, such as Cytomegalovirus, Ebola virus, Enteroviruses, Hepatitis B virus, Hepatitis C virus, Human herpesviruses, HIV, Human papillomavirus, Influenza virus, Measles virus, Rhinoviruses and Rubella virus [49, 50]. As previously mentioned, Zika and dengue virus were also discovered in saliva [30, 40]. Although, the presence of intact viral particles in saliva do not distinguish viable virus from noninfectious virus. However, for the first time, we could well identify ZIKV plaque forming units from saliva of an infected man in Vero cell monolayers with a titer corresponding to 80 PFU per ml.

Essentially, another important subject is that the existence of viable virus in oral fluid samples does not always indicate that the virus can be transmitted orally and become epidemiologically relevant. Actually, viral infections of the oral cavity are relatively rare, since saliva contains antiviral molecules and is relatively hypotonic being capable of lysing enveloped viruses [51]. Perhaps, the established proportion of approximately 1 PFU to 1,000 ZIKV RNA copies in saliva of one patient was modulated by these host factors.

Although saliva functions as a protective barrier for virus entry, some studies have shown that a disruption in oral mucosa or periodontal disease can facilitate virus entry [52]. Since previous studies detected Flaviviruses as Dengue [53, 54] and Zika [30] virus in saliva, and our study have demonstrated possible infectious ability of Zika viral particles in saliva, a potential person-to-person Zika virus infection through this specimen, using a disrupted oral mucosa or periodontal pockets as virus entry, should be considered and investigated.

ZIKV is an emergent vector-borne disease, but fast growing evidence points to an increased relevance of its non-vector ways of transmission, as perinatal and transplacental transmission occurs from mother to child [14, 23]. Additionally, ZIKV genome was also detected in breast milk, followed by viral isolation of infective viral particles [29]. Moreover, cases of probable sexual transmission have been reported with association of ZIKV in semen [25, 26]. In addition, viral contamination linked to blood transfusion and organ transplantation have been previously discussed [55]. Furthermore, reports of laboratorial infection or bites of animals was associated to the transmission [56]. Finally, evidence of vertical and/or venereal transmission between mosquitoes was supported by the detection of ZIKV natural infection in males *Ae*. *furcifer* [19].

We compared the complete coding sequences obtained in this study with public sequence data from Zika virus representative of the isolates from three distinct genotypes in Asian, West African, and East African in addition to isolates from recent outbreak in Americans. Similarly to the sequences described in the recent widespread epidemic of ZIKV in the Americas, the sequences Rio-S1 and Rio-U1 from ZIKV isolated in this study clustered with the Asian clade, covering sequences from New World, Pacific, Micronesian and Malaysian strains.

Since surveillance programs have reported periodic circulation of the ZIKV virus since 1968, with high frequency activity varying an interval of 1–2 years added to fact that RNA virus evolve fast, their host and vector broad range, non-vector transmission, and particularly risk of neurotropic and teratogenic outcomes, the molecular epidemiologic vigilance is crucial to solve this questions.

In conclusion, the detection of infective ZIKV in saliva and urine of patients deserves a more detailed study to establish whether or not these fluids contribute to viral transmission. Surely, these findings will be extremely relevant to prevent and control ZIKV transmission.

## Supporting Information

S1 Text Supporting Information contains Tables describing clinical (Table A and B) and social demographic (Table C and D) characteristics of the enrolled patients. (DOCX)

S1 Checklist STROBE checklist. (DOCX)

## References

[1] Kuno G., et al Phylogeny of the genus Flavivirus. J Virol, 1998 72(1): p. 73–83. 9420202 10.1128/jvi.72.1.73-83.1998 PMC109351

[2] Dick G.W., Kitchen S.F., and Haddow A.J., Zika virus. I. Isolations and serological specificity. Trans R Soc Trop Med Hyg, 1952 46(5): p. 509–20. 12995440 10.1016/0035-9203(52)90042-4

[3] Hayes E.B., Zika virus outside Africa. Emerg Infect Dis, 2009 15(9): p. 1347–50. 10.3201/eid1509.090442 19788800 PMC2819875

[4] Duffy M.R., et al Zika virus outbreak on Yap Island, Federated States of Micronesia. N Engl J Med, 2009 360(24): p. 2536–43. 10.1056/NEJMoa0805715 19516034

[5] Musso D., Nilles E.J., and Cao-Lormeau V.M., Rapid spread of emerging Zika virus in the Pacific area. Clin Microbiol Infect, 2014 20(10): p. O595–6. 10.1111/1469-0691.12707 24909208

[6] Cao-Lormeau V.M., et al Zika virus, French polynesia, South pacific, 2013. Emerg Infect Dis, 2014 20(6): p. 1085–6. 10.3201/eid2006.140138 24856001 PMC4036769

[7] CDC. *Zika virus*. 2016.

[8] Oehler E., et al Zika virus infection complicated by Guillain-Barre syndrome—case report, French Polynesia, December 2013. Euro Surveill, 2014 19(9). 10.2807/1560-7917.es2014.19.9.20720 24626205

[9] Zanluca C., et al First report of autochthonous transmission of Zika virus in Brazil. Mem Inst Oswaldo Cruz, 2015 110(4): p. 569–72. 10.1590/0074-02760150192 26061233 PMC4501423

[10] Campos G.S., Bandeira A.C., and Sardi S.I., Zika Virus Outbreak, Bahia, Brazil. Emerg Infect Dis, 2015 21(10): p. 1885–6. 10.3201/eid2110.150847 26401719 PMC4593454

[11] WHO, Zika virus infection: global update on epidemiology and potentially associated clinical manifestations. Wkly Epidemiol Rec, 2016 91(7): p. 73–81. 26897760

[12] Kleber de Oliveira W., et al Increase in Reported Prevalence of Microcephaly in Infants Born to Women Living in Areas with Confirmed Zika Virus Transmission During the First Trimester of Pregnancy—Brazil, 2015. MMWR Morb Mortal Wkly Rep, 2016 65(9): p. 242–7. 10.15585/mmwr.mm6509e2 26963593

[13] Saúde, M.d. INFORME EPIDEMIOLÓGICO N° 15 –SEMANA EPIDEMIOLÓGICA (SE) 08/2016 (21 A 27/02/2016) MONITORAMENTO DOS CASOS DE MICROCEFALIA NO BRASIL. 2016: Brasília, Brazil.

[14] Mlakar J., et al Zika Virus Associated with Microcephaly. N Engl J Med, 2016. 10.1056/NEJMoa1600651 26862926

[15] Calvet G., et al Detection and sequencing of Zika virus from amniotic fluid of fetuses with microcephaly in Brazil: a case study. Lancet Infect Dis, 2016. 10.1016/S1473-3099(16)00095-5 26897108

[16] Brasil P., et al Zika Virus Infection in Pregnant Women in Rio de Janeiro—Preliminary Report. N Engl J Med, 2016. 10.1056/NEJMoa1602412 PMC5323261 26943629

[17] Gatherer D. and Kohl A., Zika virus: a previously slow pandemic spreads rapidly through the Americas. J Gen Virol, 2016 97(2): p. 269–73. 10.1099/jgv.0.000381 26684466

[18] WHO. WHO Director-General summarizes the outcome of the Emergency Committee regarding clusters of microcephaly and Guillain-Barré syndrome. 2016; Available from: http://www.who.int/mediacentre/news/statements/2016/emergency-committee-zika-microcephaly/en/.

[19] Diallo D., et al Zika virus emergence in mosquitoes in southeastern Senegal, 2011. PLoS One, 2014 9(10): p. e109442 10.1371/journal.pone.0109442 25310102 PMC4195678

[20] Faye O., et al Quantitative real-time PCR detection of Zika virus and evaluation with field-caught mosquitoes. Virol J, 2013 10: p. 311 10.1186/1743-422X-10-311 24148652 PMC4016539

[21] Grard G., et al Zika virus in Gabon (Central Africa)—2007: a new threat from Aedes albopictus? PLoS Negl Trop Dis, 2014 8(2): p. e2681 10.1371/journal.pntd.0002681 24516683 PMC3916288

[22] Chouin-Carneiro T., et al Differential Susceptibilities of Aedes aegypti and Aedes albopictus from the Americas to Zika Virus. PLoS Negl Trop Dis, 2016 10(3): p. e0004543 10.1371/journal.pntd.0004543 26938868 PMC4777396

[23] Besnard M., et al Evidence of perinatal transmission of Zika virus, French Polynesia, December 2013 and February 2014. Euro Surveill, 2014 19(13). 24721538

[24] Musso D., et al Potential for Zika virus transmission through blood transfusion demonstrated during an outbreak in French Polynesia, November 2013 to February 2014. Euro Surveill, 2014 19(14). 10.2807/1560-7917.es2014.19.14.20761 24739982

[25] Musso D., et al Potential sexual transmission of Zika virus. Emerg Infect Dis, 2015 21(2): p. 359–61. 10.3201/eid2102.141363 25625872 PMC4313657

[26] Foy B.D., et al Probable non-vector-borne transmission of Zika virus, Colorado, USA. Emerg Infect Dis, 2011 17(5): p. 880–2. 10.3201/eid1705.101939 21529401 PMC3321795

[27] Gourinat A.C., et al Detection of Zika virus in urine. Emerg Infect Dis, 2015 21(1): p. 84–6. 10.3201/eid2101.140894 25530324 PMC4285245

[28] Kutsuna S., et al Two cases of Zika fever imported from French Polynesia to Japan, December 2013 to January 2014 [corrected]. Euro Surveill, 2014 19(4). 10.2807/1560-7917.es2014.19.4.20683 24507466

[29] Dupont-Rouzeyrol M., et al Infectious Zika viral particles in breastmilk. Lancet, 2016. 10.1016/S0140-6736(16)00624-3 26944028

[30] Musso D., et al Detection of Zika virus in saliva. J Clin Virol, 2015 68: p. 53–5. 10.1016/j.jcv.2015.04.021 26071336

[31] Brasil P., et al Zika Virus Outbreak in Rio de Janeiro, Brazil: Clinical Characterization, Epidemiological and Virological Aspects. PLoS Negl Trop Dis, 2016 10(4): p. e0004636 10.1371/journal.pntd.0004636 27070912 PMC4829157

[32] Hasebe F., et al Combined detection and genotyping of Chikungunya virus by a specific reverse transcription-polymerase chain reaction. J Med Virol, 2002 67(3): p. 370–4. 12116030 10.1002/jmv.10085

[33] Lanciotti R.S., et al Rapid detection and typing of dengue viruses from clinical samples by using reverse transcriptase-polymerase chain reaction. J Clin Microbiol, 1992 30(3): p. 545–51. 1372617 10.1128/jcm.30.3.545-551.1992 PMC265106

[34] Larkin M.A., et al Clustal W and Clustal X version 2.0. Bioinformatics, 2007 23(21): p. 2947–8. 17846036 10.1093/bioinformatics/btm404

[35] Nei M.a. K. S., Molecular Evolution and Phylogenetics. 2000: Oxford University Press 352

[36] Kumar S., Stecher G., and Tamura K., MEGA7: Molecular Evolutionary Genetics Analysis version 7.0 for bigger datasets. Mol Biol Evol, 2016. 10.1093/molbev/msw054 PMC8210823 27004904

[37] Tamura K., et al Estimating divergence times in large molecular phylogenies. Proc Natl Acad Sci U S A, 2012 109(47): p. 19333–8. 10.1073/pnas.1213199109 23129628 PMC3511068

[38] Lanciotti RS L.A., Holodniy M, Saavedra S, del Carmen Castillo Signor L., Phylogeny of Zika virus in Western Hemisphere, 2015 [letter]. Emerg Infect Dis., 2016. 10.3201/eid2205.160065 PMC4861537 27088323

[39] Barzon L., et al Isolation of infectious Zika virus from saliva and prolonged viral RNA shedding in a traveller returning from the Dominican Republic to Italy, January 2016. Euro Surveill, 2016 21(10). 10.2807/1560-7917.ES.2016.21.10.30159 26987769

[40] Mizuno Y., et al Confirmation of dengue virus infection by detection of dengue virus type 1 genome in urine and saliva but not in plasma. Trans R Soc Trop Med Hyg, 2007 101(7): p. 738–9. 17418320 10.1016/j.trstmh.2007.02.007

[41] Domingo C., et al Detection of yellow fever 17D genome in urine. J Clin Microbiol, 2011 49(2): p. 760–2. 10.1128/JCM.01775-10 21106799 PMC3043487

[42] Luby J.P., et al Antigenuria in St. Louis encephalitis. Am J Trop Med Hyg, 1980 29(2): p. 265–8. 6989277 10.4269/ajtmh.1980.29.265

[43] Mathur A., et al Viruria during acute Japanese encephalitis virus infection. Int J Exp Pathol, 1995 76(2): p. 103–9. 7786760 PMC1997161

[44] Tonry J.H., et al West Nile virus detection in urine. Emerg Infect Dis, 2005 11(8): p. 1294–6. 16102323 10.3201/eid1108.050238 PMC3320471

[45] Barzon L., et al Isolation of West Nile virus from urine samples of patients with acute infection. J Clin Microbiol, 2014 52(9): p. 3411–3. 10.1128/JCM.01328-14 24951801 PMC4313171

[46] Roze B., et al Zika virus detection in urine from patients with Guillain-Barre syndrome on Martinique, January 2016. Euro Surveill, 2016 21(9). 10.2807/1560-7917.ES.2016.21.9.30154 26967758

[47] Stiasny K. and Heinz F.X., Flavivirus membrane fusion. J Gen Virol, 2006 87(Pt 10): p. 2755–66. 16963734 10.1099/vir.0.82210-0

[48] Heinz F.X., et al Structural changes and functional control of the tick-borne encephalitis virus glycoprotein E by the heterodimeric association with protein prM. Virology, 1994 198(1): p. 109–17. 8259646 10.1006/viro.1994.1013

[49] Corstjens P.L., Abrams W.R., and Malamud D., Saliva and viral infections. Periodontol 2000, 2016 70(1): p. 93–110. 10.1111/prd.12112 26662485 PMC7167623

[50] Malamud D., Saliva as a diagnostic fluid. Dent Clin North Am, 2011 55(1): p. 159–78. 10.1016/j.cden.2010.08.004 21094724 PMC3011946

[51] Shugars D.C., Endogenous mucosal antiviral factors of the oral cavity. J Infect Dis, 1999 179 Suppl 3: p. S431–5. 10099113 10.1086/314799

[52] Sufiawati I. and Tugizov S.M., HIV-associated disruption of tight and adherens junctions of oral epithelial cells facilitates HSV-1 infection and spread. PLoS One, 2014 9(2): p. e88803 10.1371/journal.pone.0088803 24586397 PMC3931628

[53] Andries A.C., et al Value of Routine Dengue Diagnostic Tests in Urine and Saliva Specimens. PLoS Negl Trop Dis, 2015 9(9): p. e0004100 10.1371/journal.pntd.0004100 26406240 PMC4583371

[54] Anders K.L., et al An evaluation of dried blood spots and oral swabs as alternative specimens for the diagnosis of dengue and screening for past dengue virus exposure. Am J Trop Med Hyg, 2012 87(1): p. 165–70. 10.4269/ajtmh.2012.11-0713 22764309 PMC3391044

[55] Marano G., et al Zika virus and the never-ending story of emerging pathogens and Transfusion Medicine. Blood Transfus, 2016 14(2): p. 95–100. 10.2450/2015.0066-15 26674815 PMC4786129

[56] Leung G.H., et al Zika Virus Infection in Australia Following a Monkey Bite in Indonesia. Southeast Asian J Trop Med Public Health, 2015 46(3): p. 460–4. 26521519

