# Supplementary material for: Isolation of Infective Zika Virus from Urine and Saliva of Patients in Brazil
Source: PLoS Negl Trop Dis. 2016 Jun 24;10(6):e0004816. doi: 10.1371/journal.pntd.0004816 (PMC4920388; doi:10.1371/journal.pntd.0004816)
Supplement: S1 Text — (DOCX) [file pntd.0004816.s001.docx]

Table A

| **Clinical symptoms** | **Patient** | | | |
| --- | --- | --- | --- | --- |
|  | **1** | **2** | **3** | **4** |
| Onset date | 01/11/16 | 01/24/16 | 01/22/16 | 01/31/16 |
| Days after symptoms onset * | 3 | 2 | 5 | 1 |
| Days after rash onset * | <1** | 1 | 1 | 1 |
| Rash duration | 5 days | 6 days | 2 days | 2 days |
| Rash type | Macular | Macular and maculo-papular | Maculo-papular | Maculo-papular |
| Low grade fever  (duration) | - | +  (2 days) | +  (4 days) | - |
| Headache | - | - | + | - |
| Retro-orbital pain | - | - | + | - |
| Photophobia | - | - | NI | - |
| Fatigue/ malaise | - | + | + | - |
| Myalgia | - | + | + | - |
| Arthralgia  (region) | +  (wrist and elbows) | - | + (large and small joints) | - |
| Arthritis | - | - | - | - |
| Anorexia | - | - | + | - |
| Nausea/ vomiting | - | - | + | - |
| Diarrhea | - | - | - | - |
| Abdominal pain | - | - | - | - |
| Dysuria | - | - | - | - |
| Bleeding/ petechia | - | - | - | - |
| Dizziness/ light headedness | - | - | - | - |
| Pruritus | + | - | + | + |
| Paresthesias | + | - | - | - |
| Conjunctivitis | - | - | - | - |
| Edema  (local/site) | +  (hands) | - | - | - |
| Lymphadenopathy  (local/ site) | - | - | - | - |
| Enanthem | NI | - | NI | - |
| Respiratory symptoms | - | - | - | - |
| Jaundice | - | - | - | - |
| Seizures | - | - | - | - |

* Regarding the date of sample collection; **the collection date was in the first day of rash manifestation

Table B

| **Clinical symptoms** | **Patient** | | | | |
| --- | --- | --- | --- | --- | --- |
|  | **5** | **6** | **7** | **8** | **9** |
| Onset date | 01/26/16 | 01/26/16 | 01/31/16 | 01/31/16 | 01/28/16 |
| Days after sypmtons onset * | 2 | 3 | 1 | 2 | 5 |
| Days after rash onset * | 2 | <1 | 1 | 2 | 2 |
| Rash duration | 20 days | 4 days | 5 days | 6 days | 3 days |
| Rash type | Macular | Maculo-papular | Maculo-papular | Macular and maculo-papular | Maculo-papular |
| Low grade fever  (duration) | - | +  (1 day) | - | +  (2 days) | - |
| Headache | + | - | - | + | + |
| Retro-orbital pain | + | - | - | + | + |
| Photophobia | + | - | - | - | + |
| Fatigue/ malaise | + | + | - | + | - |
| Myalgia | + | + | - | - | + |
| Arthralgia  (region) | - | +  (wrists, ankles, knees, hands) | - | +  (large and small joints) | - |
| Arthritis | - | - | - | - | - |
| Anorexia | + | + | - | - | - |
| Nausea/ vomiting | + | - | - | - | - |
| Diarrhea | + | - | - | - | - |
| Abdominal pain | - | + | - | - | - |
| Dysuria | - | - | - | - | - |
| Bleeding/ petechia |  | - | - | - | - |
| Dizziness/ light headedness | + | - | - | - | - |
| Pruritus | + | + | + | + | + |
| Paresthesias | + | + | - | - | - |
| Conjunctivitis | + | + | - | - | + |
| Edema | +  (feet and hands) | +  (hands) | - | + | - |
| Lymphadenopathy  (local/ site) | +  (Cervical and auricular) | - | - | - | +  (cervical) |
| Enanthem | - | - | - | - | - |
| Respiratory symptoms | + | - | - | + | - |
| Jaundice | - | - | - | - | - |
| Seizures | - | - | - | - | - |

* Regarding the date of sample collection; **the collection date was in the first day of rash manifestation

Table C

| **Social-demographic data** | **Patient** | | | |
| --- | --- | --- | --- | --- |
|  | **1** | **2** | **3** | **4** |
| Gender | female | female | male | female |
| Age | 36 | 30 | 24 | 42 |
| Gestational age (weeks) | 18 | 33 | NA | 21 |
| Family members illness | - | - | + | - |
| Partner illness | - | - | NI | - |
| Repellent spray use | + | + | NI | + |
| Previous DENV infection | + | - | NI | - |
| Domicile in Rio de Janeiro State | Duque de Caxias | Nova Iguaçu | Rio de Janeiro | Duque de Caxias |

NA – not applicable; NI- not informed

Table D

| **Social demographic data** | **Patient** | | | | |
| --- | --- | --- | --- | --- | --- |
|  | **5** | **6** | **7** | **8** | **9** |
| Gender | female | male | female | female | female |
| Age | 30 | 68 | 20 | 27 | 22 |
| Gestational age (weeks) | NA | NA | 17 | 21 | 20 |
| Family members illness | - | + | - | - | - |
| Partner illness | - | - | - | - | - |
| Repellent spray use | + | - | + | + | + |
| Previous DENV infection | + | + | - | + | - |
| Domicile in Rio de Janeiro State | Rio de Janeiro | Rio de Janeiro | Rio de Janeiro | Duque de Caxias | Rio de Janeiro |

NA – not applicable; NI- not informed
