# Supplementary material for: Isolation of Infective Zika Virus from Urine and Saliva of Patients in Brazil
Source: PLoS Negl Trop Dis. 2016 Jun 24;10(6):e0004816. doi: 10.1371/journal.pntd.0004816 (PMC4920388; doi:10.1371/journal.pntd.0004816)
Supplement: S1 Checklist — (DOCX) [file pntd.0004816.s002.docx]

STROBE Statement—Checklist of items that should be included in reports of ***cohort studies***

|  | Item No | Recommendation |
| --- | --- | --- |
| **Title and abstract** | 1 | (*a*) Indicate the study’s design with a commonly used term in the title or the abstract  - Indicated by the term “patients” |
|  |  | (*b*) Provide in the abstract an informative and balanced summary of what was done and what was found  - page 2 |
| Introduction | | |
| Background/rationale | 2 | Explain the scientific background and rationale for the investigation being reported  Pages 2 and 3 |
| Objectives | 3 | State specific objectives, including any prespecified hypotheses  Author summary, pages 3 and 4 |
| Methods | | |
| Study design | 4 | Present key elements of study design early in the paper  Pages 6 and 7, METHODS, Study facilities and patients enrollment |
| Setting | 5 | Describe the setting, locations, and relevant dates, including periods of recruitment, exposure, follow-up, and data collection  Supporting information, Tables S1, S2, S3 and S4. |
| Participants | 6 | (*a*) Give the eligibility criteria, and the sources and methods of selection of participants. Describe methods of follow-up  Pages 6 and 7, METHODS, Study facilities and patients enrollment |
|  |  | (*b*) For matched studies, give matching criteria and number of exposed and unexposed  - Not applicable. |
| Variables | 7 | Clearly define all outcomes, exposures, predictors, potential confounders, and effect modifiers. Give diagnostic criteria, if applicable  Pages 6 and 7, METHODS, Study facilities and patients enrollment |
| Data sources/ measurement | 8* | For each variable of interest, give sources of data and details of methods of assessment (measurement). Describe comparability of assessment methods if there is more than one group  Not applicable |
| Bias | 9 | Describe any efforts to address potential sources of bias  Not applicable |
| Study size | 10 | Explain how the study size was arrived at  Pages 6 and 7, METHODS, Study facilities and patients enrolment and Clinical samples. |
| Quantitative variables | 11 | Explain how quantitative variables were handled in the analyses. If applicable, describe which groupings were chosen and why  Not applicable |
| Statistical methods | 12 | (*a*) Describe all statistical methods, including those used to control for confounding –  Not applicable |
|  |  | (*b*) Describe any methods used to examine subgroups and interactions  Not applicable |
|  |  | (*c*) Explain how missing data were addressed  Not applicable |
|  |  | (*d*) If applicable, explain how loss to follow-up was addressed  Not applicable |
|  |  | (*e*) Describe any sensitivity analyses  Not applicable |
| Results | | |
| Participants | 13* | (a) Report numbers of individuals at each stage of study—eg numbers potentially eligible, examined for eligibility, confirmed eligible, included in the study, completing follow-up, and analysed  Pages 6 and 7, METHODS, Study facilities and patients enrolment and Supporting information, Tables S1, S2, S3 and S4. |
|  |  | (b) Give reasons for non-participation at each stage  Not applicable |
|  |  | (c) Consider use of a flow diagram  Not applicable |
| Descriptive data | 14* | (a) Give characteristics of study participants (eg demographic, clinical, social) and information on exposures and potential confounders  Supporting information, Tables S1, S2, S3 and S4. |
|  |  | (b) Indicate number of participants with missing data for each variable of interest  Not applicable |
|  |  | (c) Summarise follow-up time (eg, average and total amount)  Not applicable |
| Outcome data | 15* | Report numbers of outcome events or summary measures over time  Not applicable |
| Main results | 16 | (*a*) Give unadjusted estimates and, if applicable, confounder-adjusted estimates and their precision (eg, 95% confidence interval). Make clear which confounders were adjusted for and why they were included  Not applicable |
|  |  | (*b*) Report category boundaries when continuous variables were categorized  Not applicable |
|  |  | (*c*) If relevant, consider translating estimates of relative risk into absolute risk for a meaningful time period  Not applicable |
| Other analyses | 17 | Report other analyses done—eg analyses of subgroups and interactions, and sensitivity analyses  Not applicable |
| Discussion | | |
| Key results | 18 | Summarise key results with reference to study objectives  Page 21, DISCUSSION |
| Limitations | 19 | Discuss limitations of the study, taking into account sources of potential bias or imprecision. Discuss both direction and magnitude of any potential bias  Not applicable |
| Interpretation | 20 | Give a cautious overall interpretation of results considering objectives, limitations, multiplicity of analyses, results from similar studies, and other relevant evidence  Pages 22, 23 and 24, DISCUSSION |
| Generalisability | 21 | Discuss the generalisability (external validity) of the study results  Page 24, DISCUSSION |
| Other information | | |
| Funding | 22 | Give the source of funding and the role of the funders for the present study and, if applicable, for the original study on which the present article is based  Not applicable |

*Give information separately for exposed and unexposed groups.

**Note:** An Explanation and Elaboration article discusses each checklist item and gives methodological background and published examples of transparent reporting. The STROBE checklist is best used in conjunction with this article (freely available on the Web sites of PLoS Medicine at http://www.plosmedicine.org/, Annals of Internal Medicine at http://www.annals.org/, and Epidemiology at http://www.epidem.com/). Information on the STROBE Initiative is available at http://www.strobe-statement.org.
